# Social Models Enhance Apes’ Memory for Novel Events

**DOI:** 10.1038/srep40926

**Published:** 2017-01-20

**Authors:** Lauren H. Howard, Katherine E. Wagner, Amanda L. Woodward, Stephen R. Ross, Lydia M. Hopper

**Affiliations:** 1 Department of Psychology, Franklin & Marshall College, Lancaster, PA, 17603, USA; 2 Lester E. Fisher Center for the Study and Conservation of Apes, Lincoln Park Zoo, Chicago, IL, 60614, USA; 3 Department of Psychology, University of Chicago, Chicago, IL, 60637, USA

## Abstract

Nonhuman primates are more likely to learn from the actions of a social model than a non-social “ghost display”, however the mechanism underlying this effect is still unknown. One possibility is that live models are more engaging, drawing increased attention to social stimuli. However, recent research with humans has suggested that live models fundamentally alter memory, not low-level attention. In the current study, we developed a novel eye-tracking paradigm to disentangle the influence of social context on attention and memory in apes. Tested in two conditions, zoo-housed apes (2 gorillas, 5 chimpanzees) were familiarized to videos of a human hand (social condition) and mechanical claw (non-social condition) constructing a three-block tower. During the memory test, subjects viewed side-by-side pictures of the previously-constructed block tower and a novel block tower. In accordance with looking-time paradigms, increased looking time to the novel block tower was used to measure event memory. Apes evidenced memory for the event featuring a social model, though not for the non-social condition. This effect was not dependent on attention differences to the videos. These findings provide the first evidence that, like humans, social stimuli increase nonhuman primates’ event memory, which may aid in information transmission via social learning.

## Introduction and Results

Human and nonhuman primates are expert social learners, able to glean information by observing the actions of conspecifics. As such, they can gain new skills and adopt local traditions, while avoiding the need to repeatedly “reinvent the wheel”. Such non-genetic transmission – the “second inheritance system”^1^ – is what underlies our rich cultural world and the behavioral traditions observed among certain nonhuman primate species (e.g., *Pan troglodytes*^2^; *Pongo pygmaeus*^3^; *Macaca fuscata*^4^; *Ateles geoffroyi*^5^). Although research with nonhuman primates has demonstrated that they learn more quickly and are more successful in their learning after observing a social model^6^, less is known about *why* social information is so potent^7^. To explore this, researchers have employed “ghost displays”^8^, which reveal whether individuals need to see a social model demonstrate actions, or if they can learn from observing the affordances of a task. Interestingly, both human and nonhuman primates often fail to learn when watching agentless ghost displays, despite observing the same mechanical information^9^.

Why do primates learn more robustly from social models than from matched ghost displays? One possibility is that ghost displays differ from social events in terms of basic saliency, resulting in low-level attentional differences. For example, ghost displays inherently provide less visual information (as they lack a physically present model), which may result in a reduction of engagement and subsequent recall. Additionally, ghost displays present self-moving objects that appear to defy the laws of physics. Given primates’ understanding of the basics of gravity, motion, and causality on objects^10^,^11^, decreased learning from ghost displays may reflect confusion about a physically implausible outcome^12^. Contrastingly, new behavioral and neural data from humans^13^ has proposed that the presence of a live model enhances memory, and the resultant recall might underlie humans’ increased imitation from socially-modeled events. In nonhuman primates, however, it is still unknown if a social model increases a subject’s attention to, or memory of, that event.

As opposed to ghost display studies requiring action reproduction as evidence of learning^8^, eye-tracking methodologies can passively capture subjects’ gaze as a measure of attention and recall^14^. However, no eye tracking studies have explored whether nonhuman primate memory is modulated by the presence (or absence) of a social agent^13^. Therefore, in a within-subjects study we measured zoo-housed apes’ (*Gorilla gorilla gorilla* and *P. troglodytes*) attention to, and memory for, social and non-social events using an eye tracker. In the social condition, subjects viewed a video of a human hand building a three-block tower, while in the non-social condition the subjects viewed a video of an inanimate mechanical claw building a three-block tower. Unlike with previous research, all events were physically plausible (i.e. the blocks were always moved by a model), thus overcoming one of the primary methodological limitations of ghost displays. Furthermore, the videos were matched for the amount of visual information provided and the complexity of the demonstrated action.

Following the video, subjects viewed side-by-side still pictures of the tower they had just seen constructed (either by a human hand or mechanical claw), and a similar, but novel, tower. By utilizing an eye-tracking system, we recorded both global and fine-grained attention with precision and without the need for training^15^. Eye-tracking measures included the average time spent attending to the familiarization video and the percent of time spent viewing each Area of Interest (AOI) during the familiarization and test phase. Preliminary analyses found no significant effect of species, trial number, testing session, or tower presentation side at test (all *p* > 0.05), therefore subsequent analyses collapsed across these factors.

**Figure 1:**
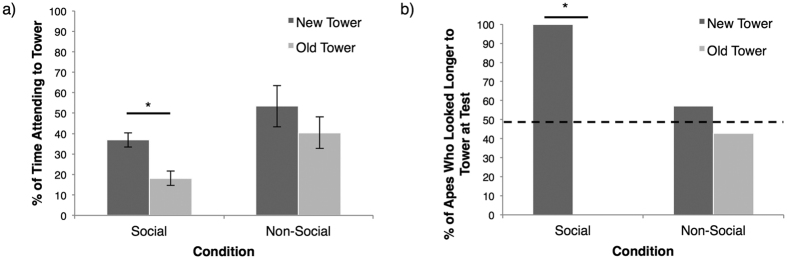
Looking time data during the memory test showing (**a**) the percent of time (number of seconds looking to tower/number of seconds attending to the screen stimuli) that apes attended to the new tower versus the old tower and (**b**) the percent of apes who demonstrated a preference for the new tower versus the old tower when compared to chance. The dotted line in (**b**) denotes chance, and *denotes *p* < 0.05.

Used extensively to explore cognitive responses in both human and nonhuman primates^16^, the looking-time method presumes that individuals will increase attention to novel versus familiar (i.e. well-remembered) stimuli after encoding an event. If a social model does increase memory for an event, apes should show a stronger memory effect (i.e. look more to the novel tower at test) in the social condition compared to in the non-social condition. On an individual level, all of the seven apes (100%) demonstrated longer looking to the new versus old tower in the social condition. A chi-square test of independence revealed that this number was significantly more than would be expected by chance (*χ*^2^(1,*N* = 7) = 7.00, *p* < 0.001, Fig. 1). In contrast, only 4/7 apes (57%) in the non-social condition evidenced a novelty preference, which was not significantly higher than would be expected by chance (*χ*^2^(1,*N* = 7) = 0.14, *p* = 0.70). Therefore, apes were significantly more likely to remember the tower-building event in the social versus non-social condition.

On a group level, a 2 (Condition: social, non-social) by 2 (Tower: old, new) repeated measures ANOVA demonstrated a significant main effect of condition (*F*(1,6) = 11.52, *p* = 0.015), revealing that subjects looked longer to both towers in the non-social condition (*M* = 46.93%, *SD* = 23.40%) than the social condition (*M* = 27.50%, *SD* = 9.23%). There were no other main effects or interactions. Due to a priori hypotheses concerning looking-time patterns within condition, paired-samples t-tests were run to examine the percent of time looking to the old versus new tower in the social and non-social condition individually. In the social condition, apes spent a significantly greater percent of time looking at the new (*M* = 36.85%, *SD* = 9.05%) versus old (*M* = 18.16%, *SD* = 9.42%) block tower at test (*t*(1,6) = 3.09, *p* = 0.02), indicating that they remembered the familiarized event (Fig. 1). However, no such looking-time difference was found in the non-social condition (new tower: *M* = 53.44, *SD* = 26.79; old tower: *M* = 40.43, *SD* = 20.30, *t*(1,6) = 0.86, *p* = 0.42). Therefore, on both an individual and group (average) level, subjects evidenced memory for the event that included the social hand constructing the towers, but not for the event that included the non-social claw.

**Figure 2:**
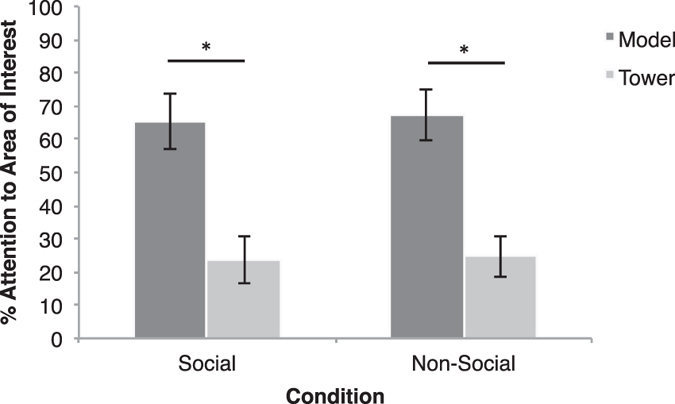
The percent of time (number of seconds looking to model/number of seconds attending to the screen stimuli) that apes attended to the model (hand or claw) and tower during the video demonstration phase. *Denotes *p* < 0.05.

The apes’ enhanced memory for social events was not dependent on differential attention while encoding the demonstration videos. A paired-samples t-test revealed no significant difference in average looking time to either the social (*M* = 7.49 seconds, *SD* = 8.57) or non-social (*M* = 6.91 seconds, *SD* = 8.67) videos during familiarization (*t*(1,6) = 0.11, *p* = 0.91), suggesting that one condition was not inherently more engaging than another. Fine-grained analyses further support these results. A 2 (Condition: social, non-social) by 2 (AOI: tower, model) repeated measures ANOVA demonstrated a significant main effect of AOI (*F*(1,6) = 30.75, *p* = 0.001): subjects looked longer to the model, social or non-social (*M* = 66.37, *SD* = 21.55), than the tower (*M* = 24.14, *SD* = 17.49; Fig. 2). However, attention to the model did not vary according to condition (*p* > 0.05). Thus, when watching the videos, subjects attended more to what was building the tower, than the tower itself, but their attention to the model did not vary according to condition.

There was a significant correlation between the apes’ attention to the tower during construction videos and their memory for the event at test in the social (*r*(7) = 0.69, *p* = 0.04, one-tailed Pearson’s correlation), but not the non-social (*p* = 0.47) condition. There was no significant correlation between the apes’ attention to the model and memory for the event in either condition (social: *p* = 0.57; non-social: *p* = 0.47). When watching videos that included a social agent, chimpanzees and gorillas that attended more to the tower being built and were also more likely to remember that tower at test. In contrast, no such correlation was found in the non-social condition.

## Discussion

Eye-tracking methods have been utilized to demonstrate long-term event memory in apes^15^, however our results are the first to demonstrate that apes’ event memory is mediated by social agents. Specifically, chimpanzees and gorillas are more likely to remember events that include social models than matched events involving inanimate objects. These findings suggest a particular role for socially-provided information on ape memory, not driven by attentional differences, which may be analogous to human children’s event memory^13^. In concert, the reported influence of a social model on human, chimpanzee and gorilla event memory suggests a foundational importance for social stimuli that appears shared among the Hominidae. Furthermore, these data provide novel insights into why, as for humans, nonhuman primate learning appears to be enhanced by seeing a social model, compared to when shown a ghost display^6^,^17^: social models aid memory for events and this memory in turn buoys imitative social learning.

Why is it that the socially-modeled event was remembered by the apes but the inanimate event was not? One defining quality of social models is their inherent agency and their ability to produce intentional goal-directed actions^18^,^19^,^20^. This agency is matched across the model and learner in the social condition, providing a relatable goal action (e.g., building a tower) that may result in deeper, more multimodal, event encoding^13^. Data from the human developmental literature support this theory. Infants are more likely to imitate the goal-directed actions of a human than an inanimate object^21^,^22^, and this rate of imitation is modulated by the extent to which infants’ own motor systems are recruited during action encoding^23^. Motor “mirroring” is also evident in nonhuman primates when observing agentive actions^24^, with differential motor activation across species potentially accounting for variation in imitative abilities^25^. Thus, the apes’ increased memory for social events may be due to an agentive match across the model and learner, and the neurocognitive structures that this match provides.

Importantly, work using neural and behavioral measures has demonstrated brain activation^24^ and learning^6^,^26^,^27^ from both heterospecific and Conspecific actions, suggesting that various models can induce agentive cognitive frameworks in apes, possibly enhanced by captive apes’ experience with humans^28^. Furthermore, previous eye-tracking paradigms^15^,^19^,^29^ have successfully utilized human models when exploring social learning and social cognition in apes (studies that report differential reactions by apes to heterospecific versus conspecific stimuli find them in relation to faces specifically^30^,^31^). Therefore, it is reasonable to assume that the memory pattern found in the current study would hold regardless of the model’s species and, if anything, the presentation of a conspecific might create an even stronger effect size. Future research exploring social learning under more ecologically valid conditions (i.e., utilizing conspecific models, testing in natural habitats) may help to further elucidate the strength of this effect in other contexts.

It is important to note that, while we found no effect of species across any of our measures, our samples sizes, particularly for the gorillas, were rather small. Since we were interested in the feasibility of passively testing the subjects while they remained in their social groups, their participation was voluntary but also dependent on the social dynamics of their group^32^. Access to the eye tracker proved easier for the chimpanzees, who were willing to watch the stimuli and then move away when their turn was over, allowing multiple group members to participate. In line with previous studies run with these species at this zoo^33^, the dominant silverback gorillas were less likely to relinquish control over the testing area, often at the exclusion of lower-ranked and female group members. In order to fully understand social memory similarities or differences across this species, it would be advantageous for future research to test a larger number of gorilla subjects, as well as other great ape species. For testing species with strong dominance hierarchies in a social setting, this may be made possible through the provision of multiple testing stations.

The importance of a social model with agency, which encourages greater learning by naïve individuals in comparison to seeing a task’s affordances in the absence of a social demonstrator, may be universal among social species as this effect is seen in insects, birds and mammals^34^,^35^,^36^. More than mechanical information or social support is needed for such learning, especially for complex tasks^8^. Although the importance of a social model has long been recognized^8^ it is only recently with the application of novel technologies that researchers have begun to understand the underlying mechanisms for this^13^,^25^. By using eye tracking methods, we were able to noninvasively test apes’ memory for social and non-social events. In line with recent results reported for children^13^, the apes showed better memory for the social events. Thus, we propose that it is apes’ memory for these social events, and not simply enhanced attention to them, that aids learning. Still unanswered, however, is how this memory translates into action and faithful replication of observed events and whether it is mediated by observers’ relationship with the expert^37^. Furthermore, although humans and nonhuman primates copy those around them, human material and social cultures are markedly more complex. What underlies this advantage in our ability to both imitate and improve upon others’ actions, and how this might relate action encoding, is yet to be revealed.

### Experimental Procedures

This study was approved by the Lincoln Park Zoo Research Committee, which is the governing body for all animal research at the institution. All methods were performed in accordance with the relevant guidelines and regulations of this committee. No modifications were made to standard animal care routines and the type and number of food rewards were approved by the zoo’s veterinary and nutrition staff.

### Subjects and Housing

All testing was voluntary on the part of the apes, which were tested in a social setting in their home enclosure. Subjects were seven great apes (2 western lowland gorillas, *Gorilla gorilla gorilla*, 5 chimpanzees, *Pan troglodytes*) living at Lincoln Park Zoo, Chicago, USA. The gorilla subjects were two males (mean age 18.0 years, *SD* = 9.9), and the chimpanzee subjects were two males and three females (mean age 21.8 years, *SD* = 6.3). One of the gorillas lived in a family group with three adult females and three infants while the second gorilla lived in an all-male group of four gorillas. The chimpanzees lived together in a social group of six. All subjects had previous experience viewing stimuli on a touch-screen monitor^38^.

All subjects were housed and tested in an indoor-outdoor enclosure at the zoo’s Regenstein Center for African Apes, which incorporated climbing structures and deep-mulch bedding, and measured 4055 m^2^ (gorilla family group), 1932 m^2^ (all-male gorilla group), and 2419 m^2^ (chimpanzee social group) respectively. The apes had outdoor access when weather conditions were appropriate (>5 °C) but all testing was conducted in their indoor enclosure. Fresh produce and primate chow were scattered twice daily throughout their exhibits as part of the typical management routine (although no such food was given *during* test sessions). The animals were not water or food deprived.

### Materials and Procedure

**Figure 3:**
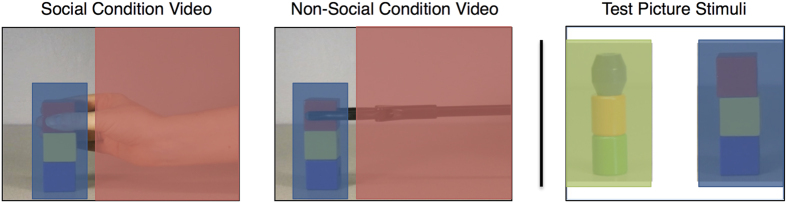
Area of Interest (AOIs) for the demonstration video and memory test picture stimuli. The shaded areas denote the AOIs for the builder (red), old tower (blue), and new tower (green). The builder (hand or claw) had a relatively large AOI to account for the fact that it entered and exited from the right side of the screen at different heights, depending on which block was being placed.

Eye gaze was collected with a Tobii x2-60 corneal reflection eye-tracking system (accuracy 0.4°, sampling rate 60 Hz) connected to an external 55 cm LCD monitor (1920 × 1080 resolution) at approximately 65 cm viewing distance. The tracking apparatus was placed on a mobile computer cart with a monitor mount. A standard 9-point human eye calibration was acquired before testing, and a single focal point was presented in the center of the screen before each test phase to assure accuracy. Data were collected and analyzed using the Tobii Studio software (Tobii Technology, Sweden). Areas of Interest (AOIs) used for analysis were drawn around the tower and the builder (hand or claw) in the demonstration video, and around the towers (old and new) for the memory test (Fig. 3).

**Figure 4:**
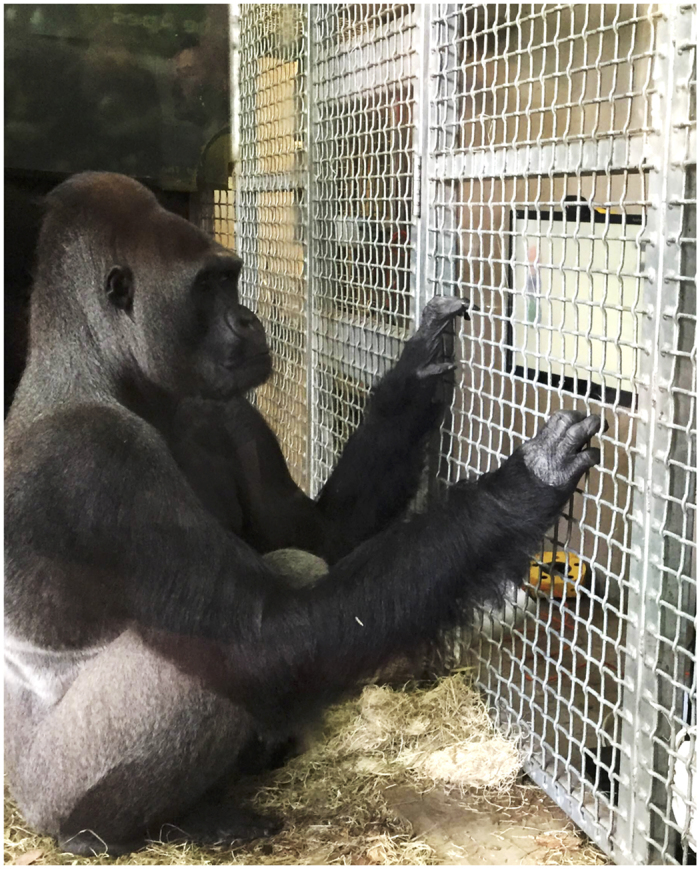
A gorilla subject attending to the tower construction video through a panel of 5 × 5 cm mesh in his home enclosure. The video stimuli was presented on a LCD monitor integrated with a Tobii x2-60 eye tracker mounted on the bottom of the screen, adjusted for the height and location of the ape. No training was provided before testing. Zoo guests were able to observe test sessions through glass viewing windows (through which this photograph was taken), and zoo educators interpreted every test session to communicate the importance of cognitive testing with the animals to zoo guests.

During testing, the apes remained with their social groups. Each animal received a small food reward (pieces of fruit) upon the completion of each session, but they were not rewarded during testing. The computer cart was pushed up against a mesh panel at the perimeter of the apes’ indoor enclosure, allowing the ape to voluntarily approach without interfering with the eye tracker (Fig. 4). Upon approach, the experimenter adjusted the height and distance of the eye tracker accordingly. After eye gaze detection, the experimenter started the tracking protocol.

**Figure 5:**
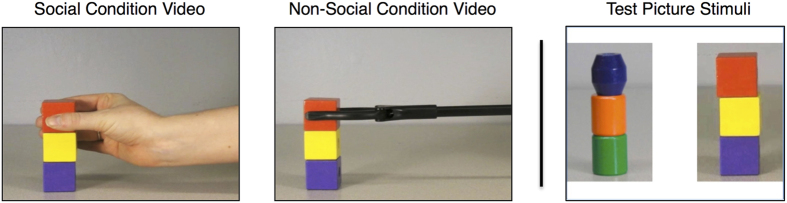
Screen capture from one of three potential test blocks depicting the demonstration videos and memory test picture stimuli seen by apes in the within-subjects social and non-social conditions. Note that each subject was presented with a unique combination of tower stimuli.

All apes were tested in both the social and non-social conditions, with testing sessions separated by a four-week delay. The order of first condition (social or non-social) was counterbalanced across subjects. Within each session, subjects viewed three test blocks, differing only in the color and shape of the objects presented across subjects. Each block started with the presentation of three 25-second long demonstration videos, showing either a social model (a human hand) or a non-social model (a mechanical-looking claw) building a three-block tower (Fig. 5). In each demonstration video, the model appeared from the right side of the screen to sequentially place three square wooden objects on the left side of the screen. Demonstration videos were filmed using a metronome set to 72 beats per minute to ensure equal speed of movement and movie length across conditions.

Following the demonstration videos, a small focal point appeared in the center of the screen for two seconds, serving as both a calibration check and attention guide. Subjects then viewed a still-picture memory test comprised of two side-by-side object towers (Fig. 5), one that was familiar (old tower) and one that was similar but novel (new tower). Each memory test appeared on the screen for five seconds. The side that the old block tower appeared on was counterbalanced across trial and participant.

Altogether, each of the three test blocks included four trials. The first trial contained three demonstration videos (to allow for adequate initial encoding of the stimuli); an attention guide, and still-picture memory test, as described above. Trials 2–4 contained a single demonstration video; an attention guide, and a memory test. Thus, all apes were given the opportunity to provide memory data for 12 test trials (four per block).

## Additional Information

**How to cite this article**: Howard, L. H. *et al*. Social Models Enhance Apes’ Memory for Novel Events. *Sci. Rep.* **7**, 40926; doi: 10.1038/srep40926 (2017).

**Publisher's note:** Springer Nature remains neutral with regard to jurisdictional claims in published maps and institutional affiliations.
